# Activated Biomass-derived Graphene-based Carbons for Supercapacitors with High Energy and Power Density

**DOI:** 10.1038/s41598-018-20096-8

**Published:** 2018-01-30

**Authors:** SungHoon Jung, Yusik Myung, Bit Na Kim, In Gyoo Kim, In-Kyu You, TaeYoung Kim

**Affiliations:** 1 Department of Bionanotechnology, Gachon University, 1342 Seongnamdaero, Seongnam, 13120 Korea; 2 Electronics and Telecommunications Research Institute, 218 Gajeong-ro, Yuseong-gu, Daejeon 34129 Korea

## Abstract

Here, we present a facile and low-cost method to produce hierarchically porous graphene-based carbons from a biomass source. Three-dimensional (3D) graphene-based carbons were produced through continuous sequential steps such as the formation and transformation of glucose-based polymers into 3D foam-like structures and their subsequent carbonization to form the corresponding macroporous carbons with thin graphene-based carbon walls of macropores and intersectional carbon skeletons. Physical and chemical activation was then performed on this carbon to create micro- and meso-pores, thereby producing hierarchically porous biomass-derived graphene-based carbons with a high Brunauer-Emmett-Teller specific surface area of 3,657 m^2 ^g^−1^. Owing to its exceptionally high surface area, interconnected hierarchical pore networks, and a high degree of graphitization, this carbon exhibited a high specific capacitance of 175 F g^−1^ in ionic liquid electrolyte. A supercapacitor constructed with this carbon yielded a maximum energy density of 74 Wh kg^−1^ and a maximum power density of 408 kW kg^−1^, based on the total mass of electrodes, which is comparable to those of the state-of-the-art graphene-based carbons. This approach holds promise for the low-cost and readily scalable production of high performance electrode materials for supercapacitors.

## Introduction

Supercapacitors or electric double layer capacitors store charges by adsorption and desorption of electrolyte ions at electrodes, as opposed to batteries which rely on electrochemical redox reactions^1–5^. This allowed supercapacitors to attain a high power density that is an order of magnitude larger than that of lithium ion batteries. Hence, supercapacitors find use in high power applications such as electronic devices and electric vehicles^2,6–8^. However, the low energy density of supercapacitors as compared to batteries hampers their widespread application. Therefore, it is imperative to develop supercapacitors with high power and energy density^4,9^. To improve the capacitance and energy density of supercapacitors, it is essential to develop carbon-based electrode materials with high specific surface area (SSA) that is easily accessible to electrolyte ions^10^. Although the commercial activated carbons (AC) are the most widely used electrode materials for supercapacitors, they suffer from low capacitance resulting from their pore architecture which may hinder ion transportation and access into the pores^11^.

In this regard, graphene is a potential material for supercapacitor electrodes because of its unique two-dimensional (2D) structure, high theoretical SSA, and high electrical conductivity^12^. Although the energy and power density of supercapacitors has been significantly improved by the use of graphene-based electrodes, their specific capacitance is still lower than the theoretical value^13^. Another reason for the limitation of the widespread use of graphene-based supercapacitor electrodes is their high cost owing to the long production time and multiple processing steps (*e.g*. oxidation of graphite into graphite oxide, exfoliation of graphite oxide into graphene oxide, reduction of graphene oxide, separation, and washing) involved in their fabrication^14^. Therefore, the development of well-designed, low-cost carbon-based materials with large accessible surface area and controllable porosity is essential for improving the performance of supercapacitors. In this context, hierarchically porous graphene-based carbons composed of thin walls of graphitic carbons and three-dimensionally interconnected pores on various length scales are considered to be promising electrode materials, since they offer the advantages of both graphene-based materials and activated carbons. While thin walls of graphitic carbons form a three-dimensional (3D) carbon framework and offer a highly conductive pathway with low intra-particle resistance, the hierarchical pore network consisting of interconnected macro- (>50 nm), meso- (2–50 nm) and micro-pores (<2 nm) ensures an efficient ion transport in the pores and the availability of large active sites for ion adsorption^15–17^. Hierarchically porous carbons have been constructed by using template-assisted methods which involve a number of synthesis steps^18^. Hard template method, for example, involves time-consuming and expensive steps such as the synthesis of a hard template, infiltration with carbon precursor, and the removal of the template^19–23^. Therefore, it is imperative to develop a simple and low-cost method for the production of hierarchically porous graphene-based carbons^24^. In this regard, abundantly available biomass such as carbohydrates (*e.g*. glucose, sucrose, fructose, starch) and lignocellulosic substances are attractive natural resources for the cost-effective production of porous graphene-like carbons^25–33^. Recent work by Bando *et. al*. illustrates that 3D porous graphene-based carbons can be made from biomass resources by the so-called sugar-blowing technique^34,35^. Despite several advances made to this method, the carbons prepared with this method have a 3D bubble network with macropores that are mostly closed or clogged. This may further require the optimization of pore architecture for efficient ion transport within the electrodes.

Herein, we present a strategy for the production of hierarchically porous graphene-based carbons from a biomass source. For the production of graphene-based carbons, glucose was gradually heated with ammonium chloride and was blown into 3D bubble network, followed by a subsequent carbonization process to form a 3D macroporous carbon framework. Hierarchical pore networks were then developed by creating small nanoscale pores in the walls of macropores through activation process using carbon dioxide (CO_2_) and potassium hydroxide (KOH). The carbons so produced consisted of thin walls of graphene layers with 3D macropores and a large volume of micro- and meso-pores. Hence, hierarchically porous biomass-derived graphene-based carbons were produced and explored as a new type of carbon electrode for supercapacitors.

## Results and Discussion

### Synthesis of activated biomass-derived graphene based carbons (a-BGC)

**Figure 1:**
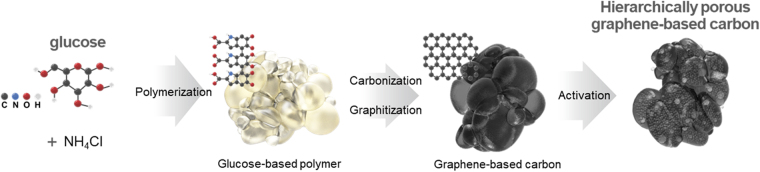
Schematic illustration of the synthesis of hierarchically porous biomass-derived graphene-based materials.

Figure 1 illustrates the method for the preparation of the hierarchically porous graphene-based carbons from a biomass source. The synthesis starts with the formation of 3D bubble networks by gradual heating of a syrup of glucose and ammonium chloride (NH_4_Cl). During heating, glucose was polymerized and blown into 3D bubbles by gases released from the decomposition reaction of ammonium chloride. Upon further heating, the glucose-based polymers in a network of bubbles was turned into 3D macroporous carbons through carbonization and graphitization^34,35^.

The resulting macroporous carbons consisted of thin walls of graphitized carbons resulting from original bubble walls and intersectional carbon skeletons. These biomass-derived graphene-based carbons (BGC) were treated to develop pores and to open the macropores that were closed, clogged, or obstructed. Both physical and chemical activation using CO_2_ and KOH were performed on the macroporous carbons. As a result, activated biomass-derived graphene-based carbons (a-BGC) were yielded with a large volume of macro-, meso-, and micropores (Table 1).

**Table 1:** Specific surface area (SSA) and total pore volume of BGC, a-BGC-1 and a-BGC-2 from activation process applied in this work.

Samples		SSA (m^2^/g)	Total pore volume (cm^3^/g)
BGC (without activation)		972	0.448
a-BGC (activated)	a-BGC-1 (CO_2_ activation)	1,865	0.877
	a-BGC-2 (CO_2_/KOH activation)	3,657	2.569

### Characterization of activated biomass-derived graphene based carbon (a-BGC)

**Figure 2:**
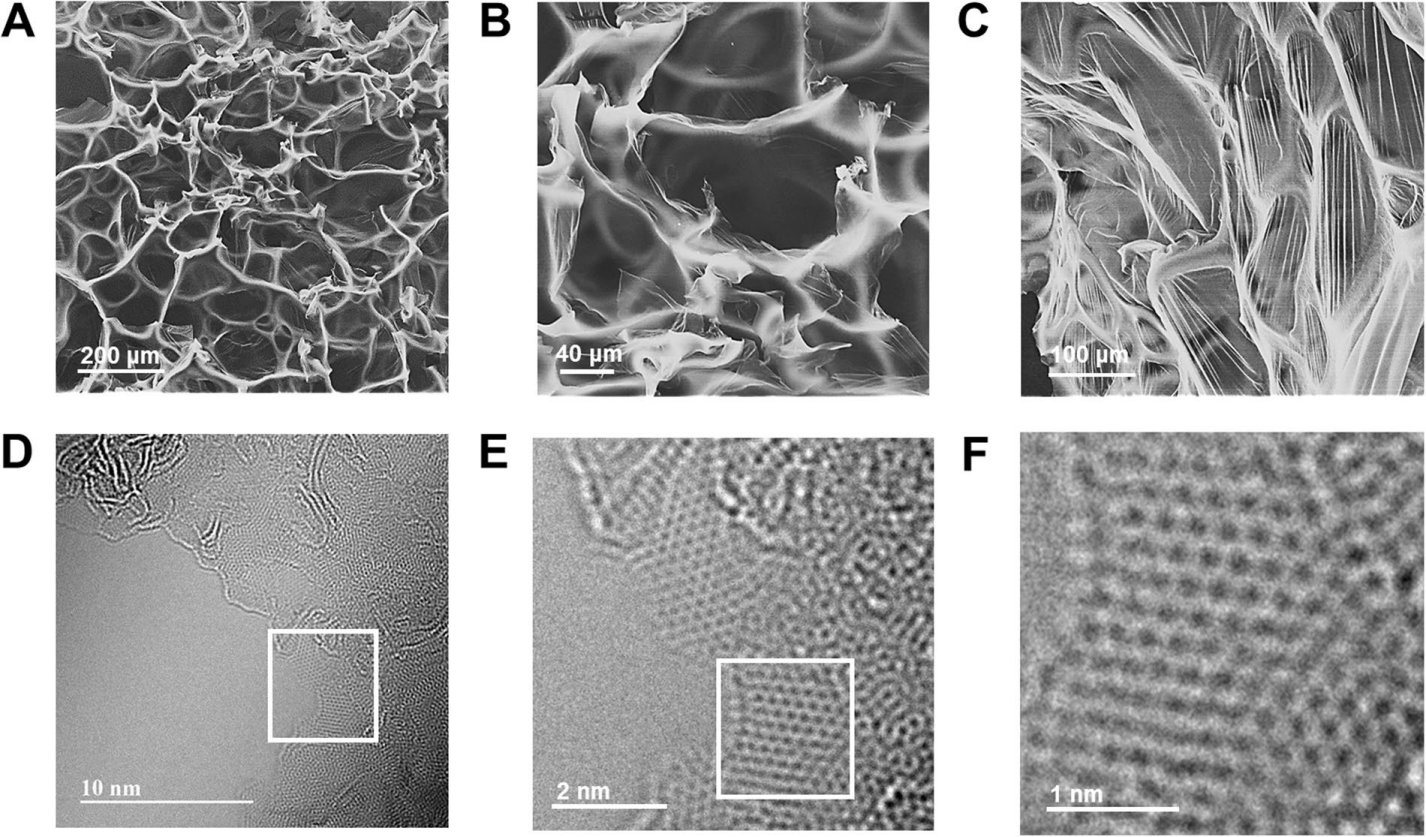
Structural and morphological characterization of a-BGC-1. (**A–C**) The SEM images with different magnification. (scale bar: 200, 40 and 100 μm, **A–C**) (**D–F**) HR-TEM images of a-BGC (scale bar: 10, 2 and 1 nm, **D–F**).

The morphology of a-BGC was examined by scanning electron microscopy (SEM). Figure 2A–C show that a-BGC-1 had a 3D macroporous framework with thin carbon walls in a network of hollow chambers and intersectional carbon skeletons. The hollow chambers surrounded by thin carbon walls has diameters ranging from 10 to 400 μm. These hollow chambers play the role of ion reservoirs and hence facilitate the efficient formation of electric double layers.

The high-resolution transmission electron microscopy (HR-TEM) results show that a-BGC was composed of crystalline carbon rings mostly arranged in a hexagonal lattice (Fig. 2D–F). The zoom-in images reveal that a large portion of the graphitized carbons was concentrated on the pore walls, thus confirming the presence of graphene layers. Unlike activated carbons which usually do not form graphene sheets even after thermal treatment at a temperature higher than 3,000 °C, the a-BGC showed a high degree of graphitization at a relatively low temperature of 1,100 °C^36^. This can be attributed to the thinning of the pore walls when the glucose-based polymers expanded to form bubbles, which promoted the development of graphitized carbon layers. As a result, the a-BGC had a large portion of graphenes on the thin wall membrane of the macropores.

**Figure 3:**
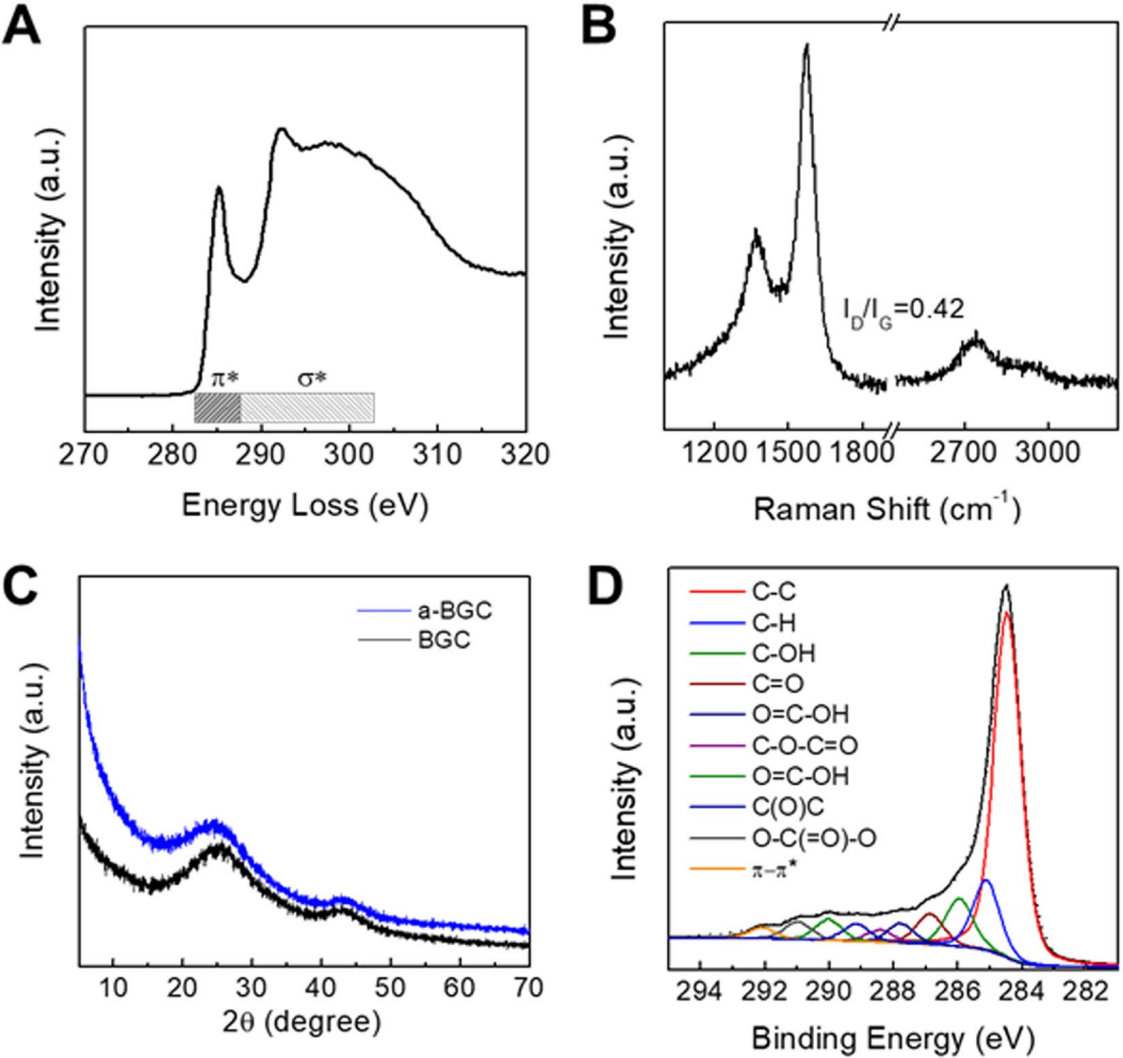
Characterization of a-BGC. (**A**) Electron energy loss spectroscopy (EELS) spectrum of a-BGC-1 (**B**) Raman spectrum of a-BGC-1, (**C**) XRD patterns of BGC and a-BGC-1 (**D**) C1s XPS spectrum of a-BGC-1.

The electron energy loss spectroscopy (EELS) spectrum of a-BGC-1 is shown in Fig. 3A. The fraction of sp^2^-bonded carbons in a-BGC was estimated by measuring the ratio between π* and π* + σ* bonding. The EELS data reveals that a-BGC showed ~99% sp^2^ bonding, which is close to that of natural graphite. The high powder conductivity of ~360 S m^−1^ measured for a-BGC-1 also supports the presence of a large fraction of sp^2^-bonded carbons. The Raman spectrum shown in Fig. 3B further reveals the existence of graphitized carbons in a-BGC. The peaks observed at 1,350 and 1,590 cm^−1^ correspond to the D (defects and disorder) and G (graphitic) bands, respectively. The intensity ratio of D- and G-bands (I_D_/I_G_) was 0.42, indicating a high graphitization degree of a-BGC. Figure 3C showed X-ray diffraction (XRD) patterns of BGC and a-BCG where (002) peaks are located at 25.9° and 25.1°, respectively. The chemical compositions of a-BGC were further investigated using X-ray photoelectron spectroscopy (XPS) (Fig. 3D**)**. The XPS survey spectrum shows that a-BGC was composed mainly of carbon atoms with a high C/O ratio of 44.5. (SI, Table S1).

**Figure 4:**
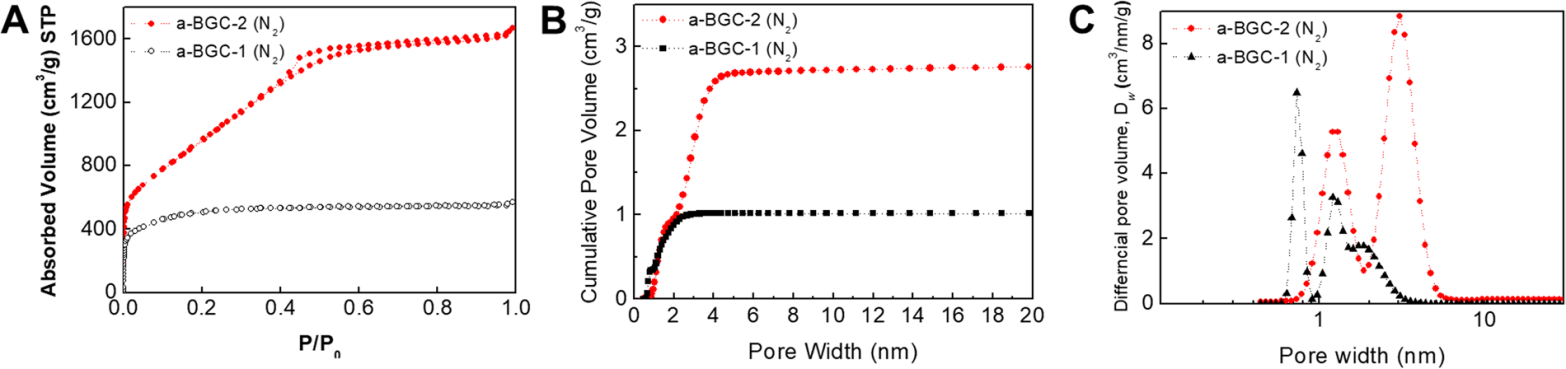
Gas sorption analysis of a-BGC-1 and a-BGC-2. (**A**) N_2_ adsorption-desorption isotherm (77 K), (**B**) Cumulative pore volume, (**C**) pore size distribution.

Figure 4 shows the N_2_ adsorption-desorption isotherms of the a-BGC samples that were activated by CO_2_ and CO_2_/KOH. While a-BGC-1 (treated by the physical activation using CO_2_) exhibited Type I behavior, a-BGC-2 (treated by the combined activation using CO_2_ and KOH) showed a Type VI behavior with H2 hysteresis loop. For both the samples, a steep increase at the low relative pressure (P/P_0_) was observed, indicating the presence of micropores in the carbons. Compared to a-BGC-1, a-BGC-2 showed an increased slope in the P/P_0_ range of 0.05–0.5, indicating that a larger volume of micro- and meso-pores were developed in this sample. This is also confirmed by the hysteresis loop between the adsorption and desorption branches, suggesting the formation of multimodal pores comprising of interconnected channels. The pore size distribution was calculated from the adsorption data and was analyzed using the nonlocal density functional theory (NLDFT)^37^, assuming a slit and cylindrical-pore geometry for the micro- and mesopores, respectively. Compared to a-BGC-1, a-BGC-2 showed a wider and multimodal distribution of pore sizes with micropores and mesopores in the 1–2 and 2–7 nm ranges, respectively.

The presence of such well-defined micro- and meso-pores in a-BGC-2 suggests the development of a hierarchical pore network, which is significant for efficient ion diffusion within the carbon electrodes. The SSA and pore volume of a-BGC were measured by the Brunauer-Emmett-Teller (BET) method using multipoint data in a P/P_0_ range of 0.1–0.3. The a-BGC-2 showed a BET SSA of 3,657 m^2 ^g^−1^, which is almost double that of a-BGC-1 (1,865 m^2 ^g^−1^), revealing that the porosity and SSA increased significantly by the combined effect of physical and chemical activation. The a-BGC-2 sample showed a large increase of up to 2.90 cm^3^ g^−1^ in the total pore volume. The high SSA and hierarchical pore networks observed in a-BGC suggest their use as electrode for supercapacitors.

## Electrochemical performance of activated biomass-derived graphene based carbons (a-BGC)

**Figure 5:**
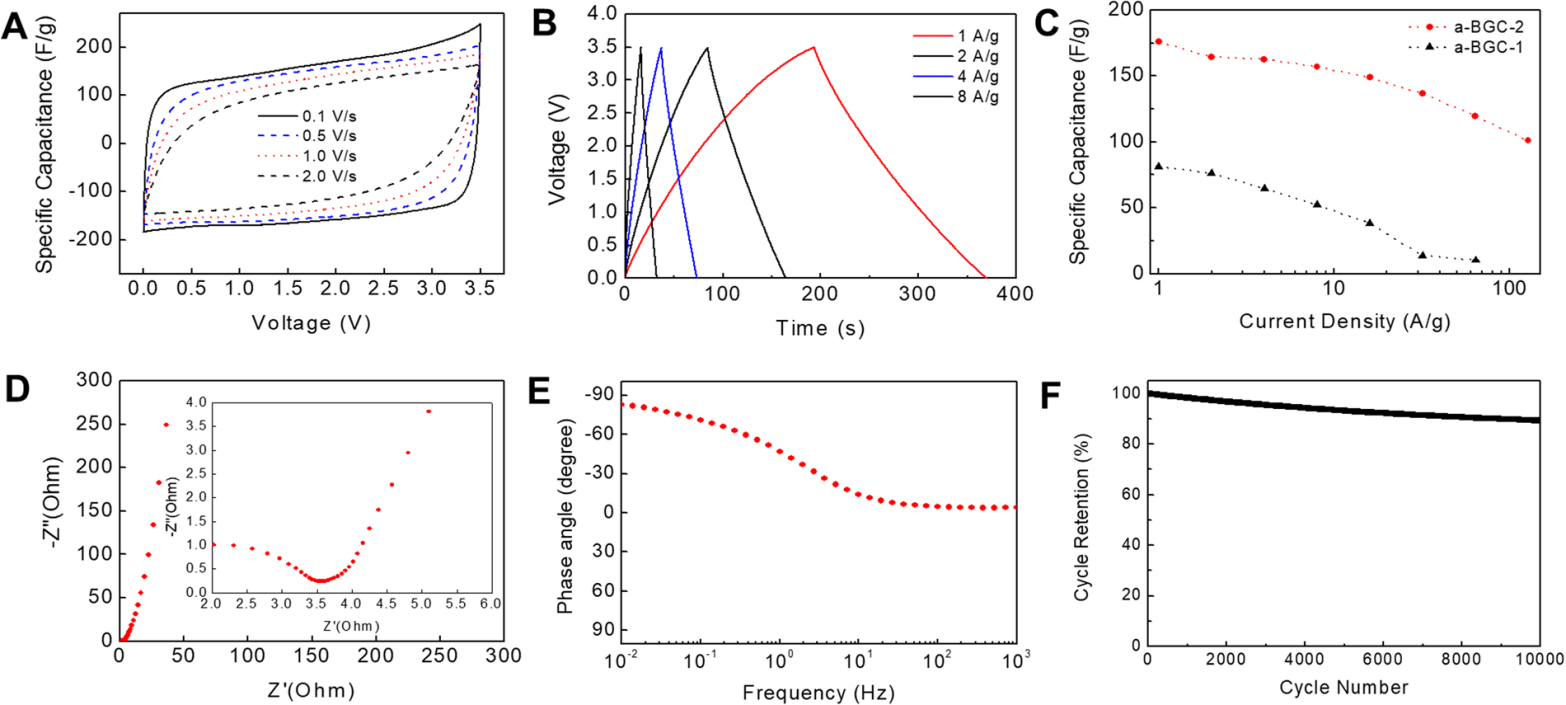
Electrochemical performance of a-BGC-2 in EMIM-TFSI/AN electrolyte. (**A**) Cyclic voltammetry (CV) profiles at different scan rates. (**B**) Galvanostatic charge-discharge curves for different current densities. (**C**) Specific capacitance at different current densities. (**D**) Nyquist plot of imaginary part and real part for impedance with a magnification for the high-frequency region in the inset. (**E**) Impedance phase angle versus frequency. (**F**) Cyclic test over 10,000 cycles.

To evaluate the electrochemical performance of a-BGC-2, we constructed a two-electrode symmetrical supercapacitor cells in organic (1 M TEA-BF_4_/AN) and ionic liquid (EMIM-TFSI/AN) electrolytes. Figure 5 show the electrochemical performance of the supercapacitors made from a-BGC-2 electrodes in ionic liquid electrolytes using cyclic voltammetry (CV) and galvanostatic charge-discharge. The CV profiles (Fig. 5A) exhibit a typical rectangular shape in the 0–3.5 V range over a wide range of voltage sweeping rates, indicating a nearly ideal capacitive behavior. Even at a high scan rate of 2 V s^−1^, the rectangular CV curves were maintained, indicating a highly reversible adsorption and desorption of the electrolyte ions onto the electrode. The high rate capability of the a-BGC-2 electrode is attributed to the hierarchical pore networks present in it and the interconnected channels developed by the activation process, giving rise to an efficient electrolyte ion movement within the electrode. The charge-discharge curves at current densities ranging from 1 to 8 A g^−1^ are shown in Fig. 5B. A good symmetry and nearly linear discharge curves also indicate a typical capacitive behavior. From the discharge curves, the specific capacitance of a-BGC was calculated to be 175, 164, 162 and 156 F g^−1^ at current densities of 1, 2, 4, and 8 A g^−1^, respectively. The a-BGC-2 sample showed the maximum specific capacitance (175 F g^−1^) in ionic liquid electrolyte at a current density of 1 A g^−1^, which is higher than those reported for biomass-derived porous carbons. (SI, Table S2).

It should be noted that BGC without activation showed a specific capacitance of ~81 F g^−1^ under the same testing conditions (SI, Figure S3), which confirms the significance of integrating micro- and meso-pores within the macroporous graphene-based carbons. Furthermore, when neat EMIM-TFSI was used as the electrolyte, the specific capacitance of a-BGC was increased up to 221 F g^−1^ (at a current density of 1 A g^−1^) (SI, Figure S4), while the equivalent series resistance (ESR) increased to ~6.6 Ω and the CV curves were not as ideal as those obtained when EMIM-TFSI/AN was used as an electrolyte. The voltage drop at the beginning of the discharge curves was 0.011 V at a current density of 1 A g^−1^, implying a low ESR of the a-BGC electrode (~4 Ω) in ionic liquid electrolytes. The electrochemical performance of a-BGC-2 was also evaluated in 1 M TEA-BF_4_/AN electrolyte (Figure S5). The CV results showed a rectangular curve and a nearly ideal capacitive characteristic over a wide range of scan rates up to 4.0 V s^−1^, indicating a high rate capability. From the discharge curve, the voltage drop was measured as 0.0067 V, suggesting a very low ESR (1.9 Ω). In Fig. 5C, a supercapacitor made from a-BGC-2 could maintain the capacitance at values higher than 100 F g^−1^, even when operated at a high charging/discharging rate of 128 A g^−1^. This result indicates the high rate capability of the device. Given the high rate capability and low voltage drop, the frequency response analysis of the a-BGC electrode in organic and ionic liquid electrolytes was carried out. Figure 5D (and SI Figure S5) shows the Nyquist plot obtained from electrochemical impedance spectroscopy (EIS) in the frequency range of 0.01 Hz–100 kHz with a magnification of the high frequency region in the inset. A vertically lined curve in the low frequency region confirms a nearly ideal capacitive behavior. Transitions from RC semicircle to the ion diffusion regime can be seen at 268 and 1,389 Hz in the EMIM-TFSI/AN and 1 M TEA-BF_4_/AN electrolytes, corresponding to the resistances of 4.0 and 1.9 Ω, respectively. The low resistance values are attributed to the fast ion diffusion in the interconnected channel of the hierarchical pore networks. The dependence of phase angle on frequency is shown in Fig. 5E. The characteristic frequency (f_0_) at a phase angle of −45° corresponds to the time constant (τ_0_ = 1/f_0_), which is the minimum time required for the full discharge of energy with an efficiency of more than 50%. The time constant was 1 and 0.5 s for the cells made up of the a-BGC electrode in the EMIM-TFSI/AN and TEA-BF_4_/AN electrolytes, respectively, indicating a high rate capability. The cycling test (Fig. 5F and SI Figure S5) showed that the cell retained ~90% of its initial capacitance after 10,000 cycles at a current density of 1 A g^−1^, indicating a good cycling stability.

**Figure 6:**
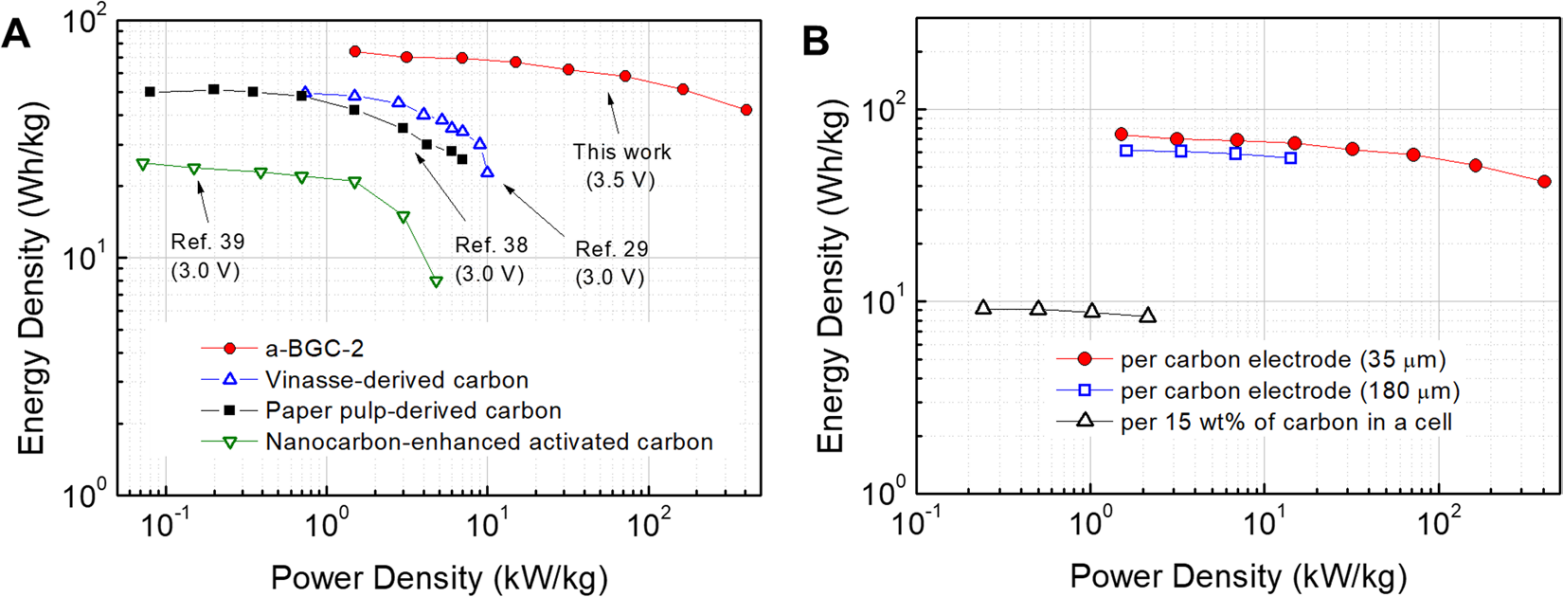
Ragone plot of the supercapacitor with a-BGC-2. (**A**) Energy and power densities of a supercapacitor with a-BGC-2 electrodes and their comparison with the reported values. (**B**) Energy and power densities normalized to the mass of the cell (black color) and active materials (red and blue color).

The overall performance of the supercapacitor made up of a-BGC electrodes is shown in the Ragone plot (Fig. 6**)**. At a specific capacitance of 175 F g^−1^ and an operating voltage of 3.5 V, the supercapacitor with a-BGC electrode and EMIM-TFSI/AN electrolyte exhibited a maximum energy density of 74 Wh kg^−1^ with a power density of 1.5 kW kg^−1^, which is higher than those with previously reported biomass-derived carbons^29,38,39^. The maximum power density was obtained at 408 kW kg^−1^, with an energy density of 42 Wh kg^−1^.

To obtain a more realistic energy and power density of the packaged cell, we assumed the a-BGC electrodes with thickness of 180 μm and electrode density of ~0.25 g cm^−1^ accounts for ~15% of the total mass of the packaged cell. That is, a factor of 6 to 7 was used to extrapolate the energy and power density of the packaged cell from the performance of our material. Energy density of ~9.1 Wh kg^−1^ was obtained for these packaged cells, which is comparable to or higher than those of supercapacitors made up of activated biomass-derived carbon electrodes. The normalized power density was as high as ~2.1 kW kg^−1^, which is higher than that of commercial supercapacitors.

## Conclusions

In summary, hierarchically porous graphene-based carbons were produced from a biomass carbon source by carbonization and graphitization of biomass-derived 3D macroporous carbons and a subsequent activation process. The resulting carbons consisted of thin graphitic carbon walls surrounding the macropores and carbon skeletons. The activation produced a large number of micro- and meso-pores within the 3D macroporous carbon frameworks, yielding hierarchically porous graphene-based carbons with a very high SSA of 3,657 m^2 ^g^−1^. This carbon exhibited a high specific capacitance of 175 F g^−1^ in an ionic liquid electrolyte with high rate capabilities. These results suggest the significance of integrating micro- and mesopores into macroporous carbon scaffolds for efficient ion transport and electric double layer formation over a large surface area. A supercapacitor constructed with this carbon yielded a maximum energy density of 74 Wh kg^−1^ and a maximum power density of 408 kW kg^−1^, which are comparable to those of the state-of-the-art carbon electrodes. The processes described herein are readily scalable to industrial level, and thus hold promise for the low-cost production of electrodes with excellent electrochemical performance.

## Methods

### Material preparation

Hierarchically porous biomass-derived graphene-based carbons were prepared by the following steps: (i) the formation and transformation of glucose-based polymers into a 3D foam-like structure, (ii) carbonization and graphitization into a macroporous graphene-based carbon, and (iii) the physical and chemical activation of a macroporous carbon to produce a hierarchically porous graphene-based carbon with an interconnected micro-, meso-, and macropores. The first step involved the formation of 3D bubble networks by heating up a syrup of glucose and ammonium chloride (NH_4_Cl) (the ratio of glucose to NH_4_Cl was 1:1 by weight) to 400 °C under an argon (Ar) flow at a heating rate of 4 °C/min. During heating, glucose was polymerized and blown into 3D bubbles by ammonia and hydrogen chloride gas released from the decomposition reaction of the ammonium chloride. Next, the glucose-based polymers in a network of bubbles were further heated under Ar gas or a mixed gas (Ar 100 sccm/CO_2_ 5 sccm) flow to 1,100 °C at a heating rate of 20 °C/min for carbonization and physical activation. The resulting product was then subjected to a chemical activation with potassium hydroxide (KOH) at a carbon:KOH weight ratio of 1:13. The carbon/KOH mixture was heated to 800 °C at a rate of 5 °C/min in an Ar atmosphere and the reaction was carried out for 1 h. The resulting product was washed with 10% acetic acid to remove the potassium salts and distilled water until a pH of 7 was obtained.

### Material Characterization

Scanning electron microscopy (SEM) images were collected on a FEI Quanta 450 FEG operated at 30 kV. The high-resolution transmission electron microscopy (HR-TEM, Titan G2 60–300, FEI, USA) operated at 80 kV was used to examine the microstructure of a-BGC. The Raman spectra were obtained on a Renishaw using 514 nm laser excitation. X-ray diffraction pattern (XRD) was recorded on PANalytical, X’pert-pro MPD, Netherlands. Elemental composition of the samples was analyzed by X-ray photoelectron spectroscopy (XPS) (Thermo Scientific, K Alpha + , U.K) with a monochromatic Al K_α_ source. Electron energy loss spectroscopy (EELS) was carried out in a TEM. Porosity was studied using N_2_ adsorption and desorption isotherms measured at 77 K on BELSORP-max (Microtrac BEL Corp., Japan). Prior to the N_2_ adsorption measurement, the samples were degassed at 110 °C under vacuum for 12 h. The SSA of the samples was calculated using the Braunauer-Emmett-Teller (BET) method in the relative pressure (P/P_0_) range of 0.1–0.3. Nonlocal density functional theory (NLDFT) was used to determine the pore size distribution.

### Electrochemical Characterization

A supercapacitor was tested in a symmetrical two electrode cell configuration. A test fixture consists of two stainless steel plates and the two electrode assembly is made of two current collectors (carbon-coated Al foil), two carbon electrodes, and a porous separator (Celgard 3501). The electrode was prepared by mixing a-BGC with poly(tetrafluoroethylene) (PTFE, 60 wt% water dispersion, Sigma Aldrich) at a a-BGC:PTFE weight ratio of 95:5. The mixture was rolled to thicknesses of ~ 35 and 180 μm, punched (10 mm diameter), and was dried in an oven at 100 °C for 24 h. The mass of the electrode was 0.7 mg and the apparent density of the electrode was 0.25 g cm^−3^. 1-ethyl-3-methylimidazolium bis(trifluoromethylsulfonyl)imide (EMIM-TFSI) diluted in acetonitrile (AN) with a weight ratio of 1:1 was used as an electrolyte. Cyclic voltammetry (CV), galvanostatic charge-discharge and electrochemical impedance spectroscopy (EIS) were examined using Potentiostat (Autolab). The specific capacitance, *C*_*sp*_ (F g^−1^) was calculated from the galvanostatic charge-discharge curves according to the equation (1).

1 $${{\rm{C}}}_{sp}=\frac{2I}{dV/dt}\times \frac{1}{m}$$

where *m (g)* is the mass of a single electrode, *I (A)* is the constant current, and *dV/dt* ${\rm{dV}}/\text{dt}\,$was obtained by linear fitting from *V*_*max*_ to 1/2*V*_*max*_ $1/2{{\rm{V}}}_{{\rm{\max }}}\,$on the discharge curves.The energy density, E (Wh kg^−1^) was calculated using the equation (2)

2 $${\rm{E}}=\frac{1}{8}{C}_{sp}{{V}_{max}}^{2}\times \frac{1}{3.6}$$

The power density, P (kW kg^−1^) was calculated using the equation (3)

3 $${\rm{P}}=\frac{E\times 3600}{{\rm{\Delta }}t}$$

where *Δt (s)* is the discharge time.

### Data availability statement

The datasets generated during and/or analyzed during the current study are available from the corresponding author on reasonable request.

## Electronic supplementary material

Supplementary Information
