# Supplementary material for: Activated Biomass-derived Graphene-based Carbons for Supercapacitors with High Energy and Power Density
Source: Sci Rep. 2018 Jan 30;8:1915. doi: 10.1038/s41598-018-20096-8 (PMC5789836; doi:10.1038/s41598-018-20096-8)
Supplement: Supplementary file 1 — Supplementary Information [file 41598_2018_20096_MOESM1_ESM.pdf]

Supplementary Information

Activated Biomass-derived Graphene-based Carbons for Supercapacitors with High Energy and Power Density

*SungHoon Jung,[1] Yusik Myung,[1] Bit Na Kim,[2] In Gyoo Kim,[2] In-Kyu You,[2] TaeYoung Kim[1]**

[1]Department of Bionanotechnology, Gachon University, Seongnamdaero 1342, Seongnam 13120, Korea

[2]Electronics and Telecommunications Research Institute, Daejeon 34129, Korea

* Corresponding Author. E-mail address: taeykim@gachon.ac.kr (TaeYoung Kim)

Table S1. Atomic percent and atomic ratio of BGC, a-BGC-1 and a-BGC-2.

	Atomic Percent (%)			Atomic Ratio (C/O)
	C	O	N	
BCG	92.78	3.62	3.60	25.60
a-BGC-1	94.49	3.16	2.35	29.90
a-BGC-2	97.80	2.20	-	44.50

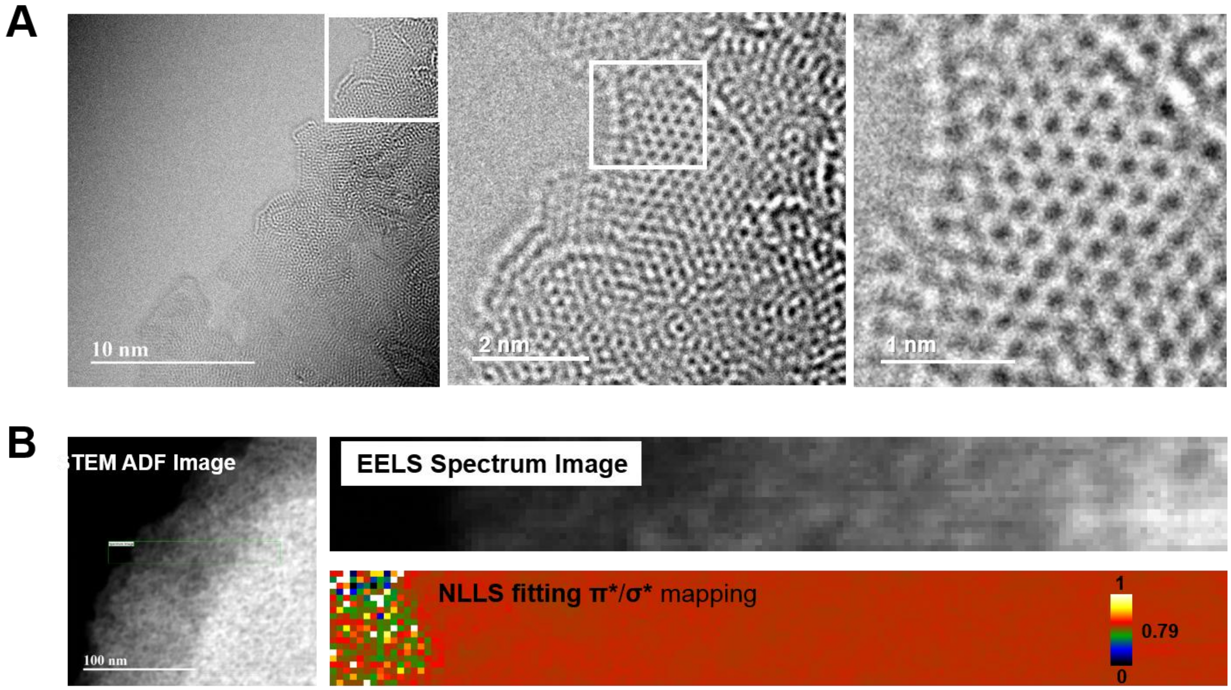

Figure S1. (A) High-resolution of TEM image of a-BGC-1 (scale bar: 10, 2 and 1 nm). (B) NLLS fitting of a-BGC-1.

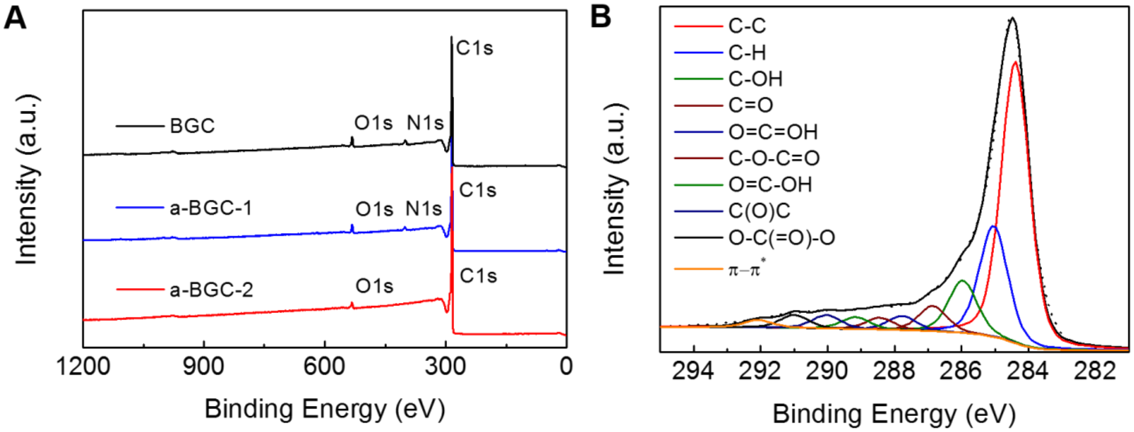

Figure S2. XPS spectrum for BGC and a-BGC. (A) Survey scan of BGC, a-BGC-1 and a-BGC-2. (C) C1s XPS spectrum of a-BGC-1.

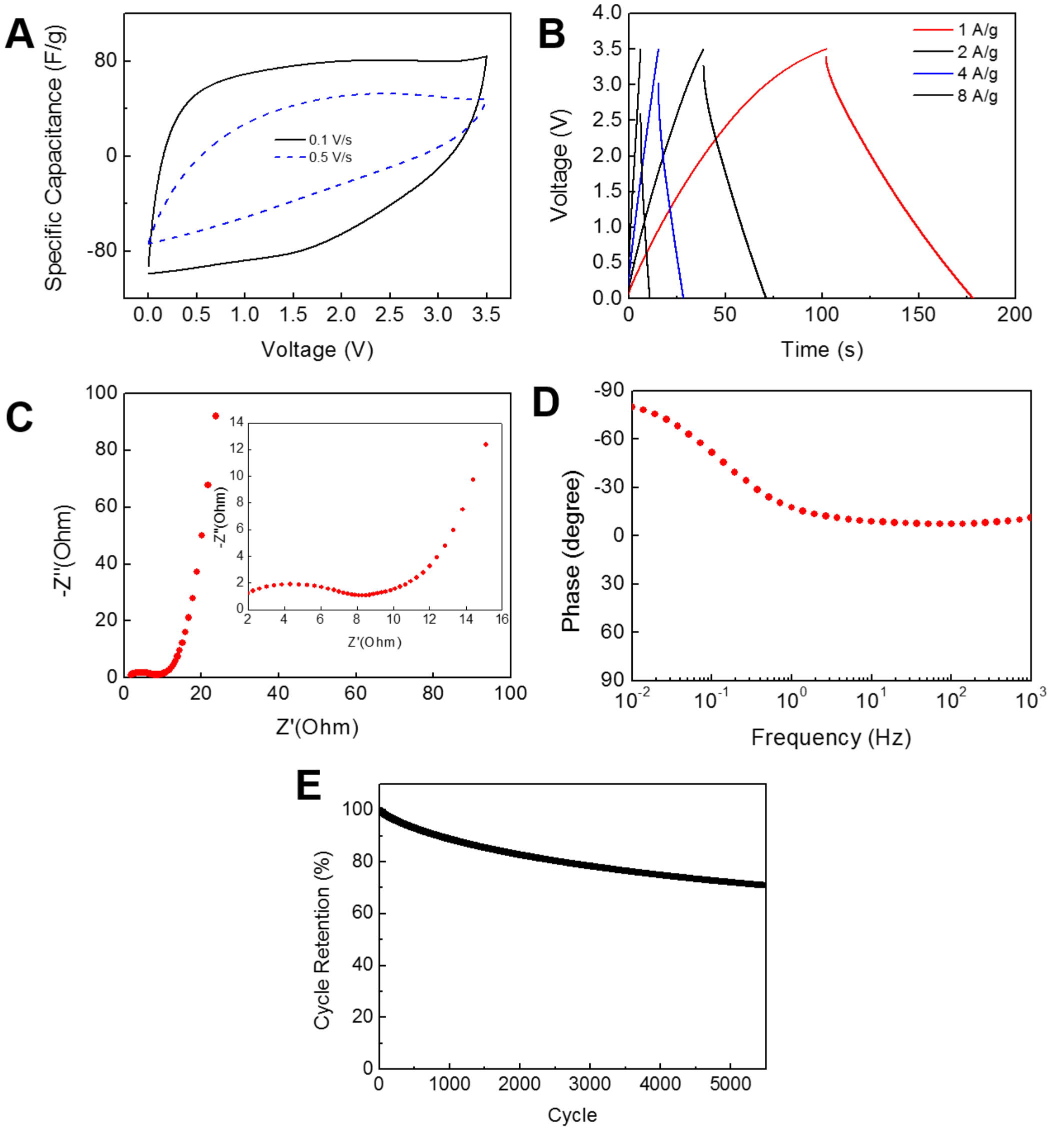

Figure S3. Electrochemical test of BGC electrode with EMIM-TFSI/AN electrolyte. (A) Cyclic voltammetry profiles at different scan rates (B) Galvanostatic charge-discharge curves for different current densities (C) Nyquist plot (D) Impedance phase angle versus frequency (E) Cyclic test over 5,000 cycles.

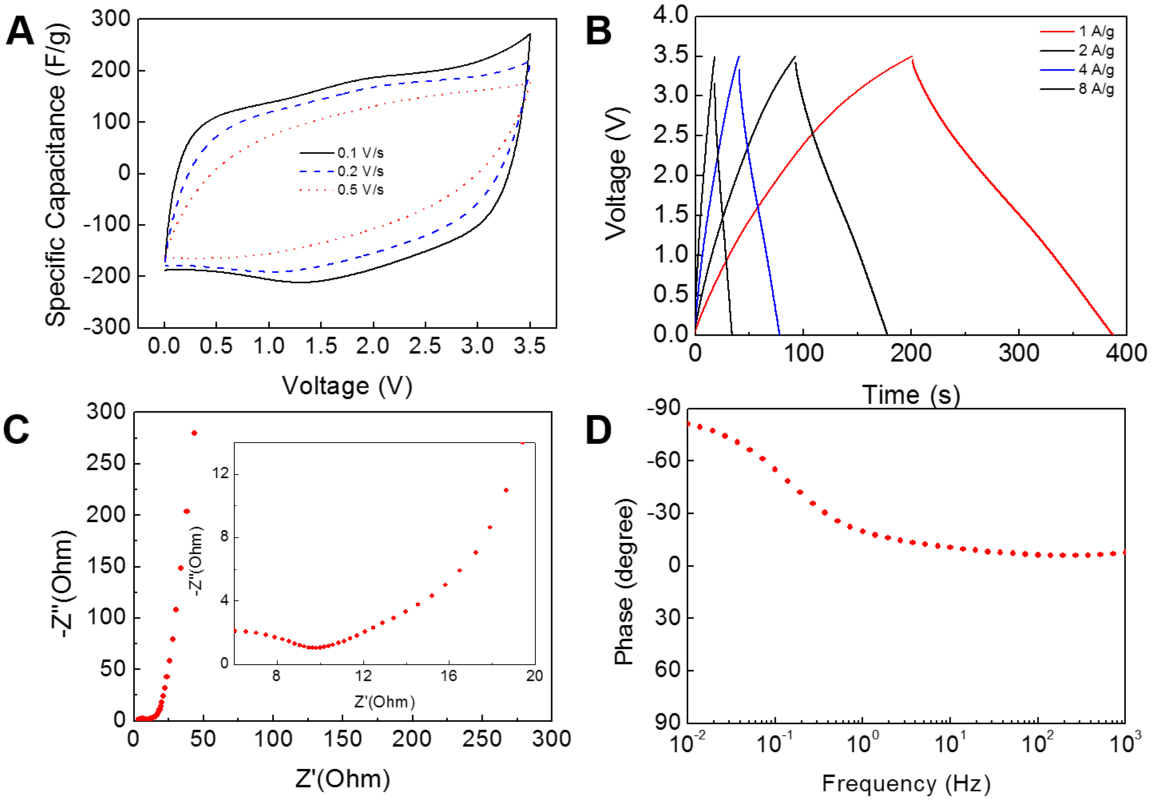

Figure S4. Electrochemical test of a-BGC-2 electrode with neat EMIM-TFSI. (A) Cyclic voltammetry profiles at different scan rates (B) Galvanostatic charge-discharge curves for different current densities (C) Nyquist plot (D) Impedance phase angle versus frequency

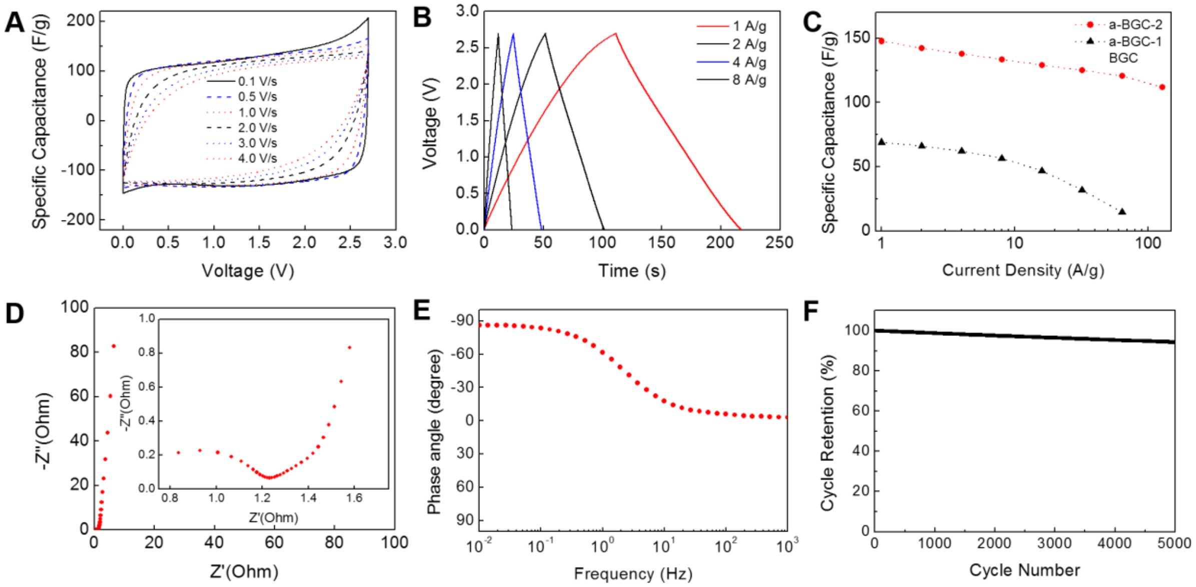

Figure S5. Electrochemical test of a-BGC-2 in 1 M $TEABF_4$/AN. (A) Cyclic voltammetry profiles at different scan rates. (B) Galvanostatic charge-discharge curves for different current densities. (C) Specific capacitance at different current densities (D) Nyquist plot (E) Impedance phase angle versus frequency (F) Cyclic test over 5,000 cycles.

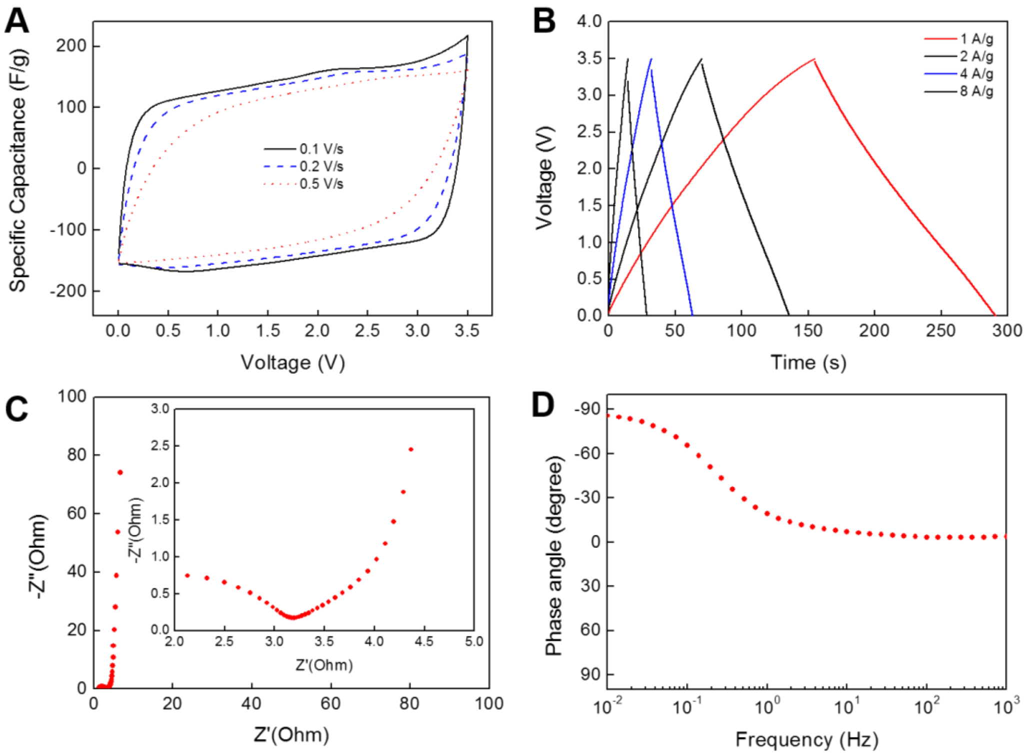

Figure S6. Electrochemical test of high mass loaded electrode (~180 μm of thickness). (A) Cyclic voltammetry profiles at different scan rates (B) Galvanostatic charge-discharge curves for different current densities (C) Nyquist plot (D) Impedance phase angle versus frequency

Table S2. Comparison of the performance of a-BGC with biomass-derived carbons reported in the literature.

Carbon Material	Specific Capacitance (F g^{-1})	Electrolyte	Ref
a-BGC	175 (1 A g^{-1}), 100 (128 A g^{-1})	EMIM-TFSI / AN	This Work
	147 (1 A g^{-1}), 111 (128 A g^{-1})	1 M $TEABF_4$ / AN	
	221 (1 A g^{-1})	EMIM-TFSI	
Vinasse-derived carbon	163 (1 A g^{-1}), 141 (20 A g^{-1})	1 M $TEABF_4$ / AN	1
Paper pulp-derived carbon	162 (0.1 A g^{-1}), 120 (10 A g^{-1})	$TEABF_4$ / AN	2
	162 (0.1 A g^{-1}), 95.3 (10 A g^{-1})	EMIM-TFSI	
Nanocarbon-enhanced activated carbon film	88 (0.1 A g^{-1})	BMPY-TFSI	3
Graphene hybrid activated carbon	196 (1 A g^{-1})	EMIM-BF_4	4
Dead leaved-derived carbon	88 (2 A g^{-1})	1 M $LiPF_6$ in EC-DEC	5

References

1. Shi, C.; Hu, L.; Guo, K.; Li, H.; Zhai, T., Highly Porous Carbon with Graphene Nanoplatelet Microstructure Derived from Biomass Waste for High-Performance Supercapacitors in Universal Electrolyte. *Advanced Sustainable Systems* **2017,** *1* (1-2).

2. Wang, H.; Li, Z.; Tak, J. K.; Holt, C. M.; Tan, X.; Xu, Z.; Amirkhiz, B. S.; Harfield, D.; Anyia, A.; Stephenson, T., Supercapacitors based on carbons with tuned porosity derived from paper pulp mill sludge biowaste. *Carbon* **2013,** *57*, 317-328.

3. Li, Z.; Liu, J.; Jiang, K.; Thundat, T., Carbonized nanocellulose sustainably boosts the performance of activated carbon in ionic liquid supercapacitors. *Nano Energy* **2016,** *25*, 161-169.

4. Huang, J.; Wang, J.; Wang, C.; Zhang, H.; Lu, C.; Wang, J., Hierarchical porous graphene carbon-based supercapacitors. *Chemistry of Materials* **2015,** *27* (6), 2107-2113.

5. Biswal, M.; Banerjee, A.; Deo, M.; Ogale, S., From dead leaves to high energy density supercapacitors. *Energy & Environmental Science* **2013,** *6* (4), 1249-1259.
